# Clinical Endoscopic Submucosal Dissection of Trainees Tutored by Experts—ESGE Endorsed Courses and Live Endoscopic Events 2011–2015

**DOI:** 10.3390/jcm15020675

**Published:** 2026-01-14

**Authors:** Daniel Neureiter, Naohisa Yahagi, Tsuneo Oyama, Takashi Toyonaga, Tobias Kiesslich, Andrej Wagner, Franz Ludwig Dumoulin, Alexander Ziachehabi, Hans-Peter Allgaier, Michael Anzinger, Gerhard Kleber, Hans Seifert, Alberto Herreros de Tejada, Ingo Steinbrück, Barbara Tribl, Alberto Tringali, Josef Holzinger, Alanna Ebigbo, João Santos-Antunes, Juergen Hochberger, Sergey V. Kantsevoy, Mathieu Pioche, Thierry Ponchon, Frieder Berr, ESD Tutoring Training Group

**Affiliations:** 1 Institute of Pathology, University Hospital, Paracelsus Medical University, 5020 Salzburg, Austria; d.neureiter@salk.at; 2 Division of Research and Development for Minimally Invasive Treatment, Cancer Center, School of Medicine, Keio University, Shinjuku-ku, Tokyo 160-8582, Japan; yahagi.keio@gmail.com; 3 Department of Endoscopy, Saku Central Hospital Advanced Care Center, Saku 3400-28, Japan; oyama@coral.ocn.ne.jp; 4 Department of Endoscopy, Kobe University Hospital, Kobe 650-0017, Japan; toyonaga@bf7.so-net.ne.jp; 5 Department of Medicine I, University Hospital, Paracelsus Medical University, 5020 Salzburg, Austria or tobias.kiesslich@pmu.ac.at (T.K.); andrej.wagner@kliniken-sob.de (A.W.); 6 Center for Physiology, Pathophysiology and Biophysics, Institute of Physiology and Pathophysiology, Paracelsus Medical University, 5020 Salzburg, Austria; 7 Department of Medicine-Gastroenterology, Klinikum SOB, 83435 Bad Reichenhall, Germany; 8 Innere Medizin/Gastroenterologie, Gemeinschaftskrankenhaus Bonn, Prinz Albert Str. 40, 53113 Bonn, Germany; f.dumoulin@gk-bonn.de; 9 Department of Medicine IV, Ordensklinikum Linz—Elisabethinen, 4020 Linz, Austria; alexander.ziachehabi@ordensklinikum.at; 10 Department of Medicine, Evangelisches Diakoniekrankenhaus, 79110 Freiburg, Germany; allgaier@diak-fr.de (H.-P.A.); ingo.steinbrueck@diak-fr.de (I.S.); 11 Department of Medicine I, Krankenhaus der Barmherzigen Brüder, 80639 Munich, Germany; michael.anzinger@barmherzige-muenchen.de; 12 Department of Medicine I, Ostalb Klinikum Aalen, 73428 Aalen, Germany; kl526489@googlemail.com; 13 Department of Medicine–Gastroenterology, University Hospital Oldenburg, 26129 Oldenburg, Germany; hansseifert@web.de; 14 Department of Gastroenterology, Puerta de Hierro University Hospital, 28222 Madrid, Spain; albertoherreros@yahoo.com; 15 Department of Gastroenterology, Asklepios Klinikum, Hamburg-Altona, 22763 Hamburg, Germany; 16 Department of Medicine III, University Hospital AKH Wien, 1090 Vienna, Austria; barbara.tribl@meduniwien.ac.at; 17 Endoscopia Digestiva, Ospedale Metropolitano Niguarda, 20162 Milan, Italy; albtri10@gmail.com; 18 Division Endoscopy, Department of Surgery, University Hospital, Paracelsus Medical University, 5020 Salzburg, Austria; j.holzinger@salk.at; 19 Department of Medicine, St Josef Hospital, Ruhr University Bochum, 44801 Bochum, Germany; alanna.ebigbo@klinikum-bochum.de; 20 Department of Gastroenterology, University Hospital São João, 4200-319 Porto, Portugal; joao.claudio.antunes@gmail.com; 21 Department of Medicine-Gastroenterology, Charité Universitätsmedizin, 13353 Berlin, Germany; juergen.hochberger@charite.de; 22 Institute for Digestive Health and Liver Disease, Mercy Medical Center, University of Maryland, Baltimore, MD 21202, USA; skan51@hotmail.com; 23 Gastroenterology and Endoscopy Unit, Edouard Herriot Hospital, Hospices Civils de Lyon, 5 Pl. d’Arsonval, 69003 Lyon, France; mathieu.pioche15@gmail.com (M.P.); thierry.ponchon@lyon.unicancer.fr (T.P.)

**Keywords:** ESD clinical training, ESD tutoring outcome, ESD indication, early GI cancer, colorectal ESD

## Abstract

**Background/Objectives**: Endoscopic submucosal dissection (ESD) is a state-of-the-art en bloc resection for early gastro-intestinal cancers and precursors developed and validated in Japan. Western expertise with this complex technique remains limited. Tutored training might be optimal for patients and ESD learning. We established ESD tutoring courses led by experienced Japanese experts to provide (i) optimal long-term curative outcomes and low complication rates for patients and (ii) hands-on training on difficult lesions for European endoscopists under direct expert supervision. **Methods**: Prospective data from 2011 to 2015 (follow-up to 12/2024) were analyzed. A total of 118 neoplasms (50% HGIEN and cancer) in 101 patients (median age 68 [37–91] years; 38% with significant comorbidities) were treated with expert or tutored ESD. Japanese experts performed 28 ESDs, while 22 trained beginners conducted 90 supervised procedures on difficult lesions during 5 live and 20 tutoring events (1–4 days each). **Results**: Analysis of the complete data showed curative and en bloc resection rates of 88% and 95%, respectively, with no recurrence after R0 resections during a median follow-up of 9.8 [1.5–14.9] years. Long-term survival remained recurrence-free after endoscopic resection of 3 recurrent adenomas (at R1/Rx) and curative surgery/2nd ESD for 5 non-curative ESDs. Adverse events occurred in 9.3% without emergency surgery or 30-day mortality. Comparing expert-only vs. tutored ESD procedures, beginners correctly applied curative ESD indications in 94% of 118 neoplasms. Experts resected larger lesions (22 cm^2^) at a rate of 9.3 cm^2^/h in 121 min. Tutored beginners achieved a 75% [25–100] self-completion rate on 33% smaller lesions in 112 min. **Conclusions**: ESD tutoring courses led by Japanese experts ensure excellent patient outcomes and standardized procedural training. This model may foster professional ESD performance across European referral centers.

## 1. Introduction

Endoscopic submucosal dissection (ESD), developed before 2010 for curative en bloc resection of early pre-/malignant neoplasms, was introduced and validated by its Japanese pioneers, who later trained interventional endoscopists [1,2] and established national ESD centers [3,4]. ESD is now the state-of-the-art oncologic en bloc resection for early GI cancers and has been adopted in Western guidelines [5,6,7,8,9,10,11]. Supervised experimental training by Japanese experts enabled Western endoscopists to begin performing ESD independently [12,13]. However, ESD demands proficiency in two learning curves: precise optical diagnosis to determine appropriate indications and advanced electrosurgical endoscopic skills [9]. Although Western endoscopists may train in Japan, due to legal restrictions, they cannot perform ESD on patients there. To address this, Naohisa Yahagi and Tsuneo Oyama—key founders of ESD—promoted an ESD tutoring program for beginners. The program was held in Salzburg (Austria) from 2011 to 2015 and remains unique in Western countries. Its goals were both to improve patient outcomes (primary objective) and simultaneously to teach accurate optical diagnosis and technical performance (secondary objectives). Here, we report the 10-year follow-up.

## 2. Materials and Methods

### 2.1. Organization and Accreditation of ESD Tutoring

**Figure 1:**
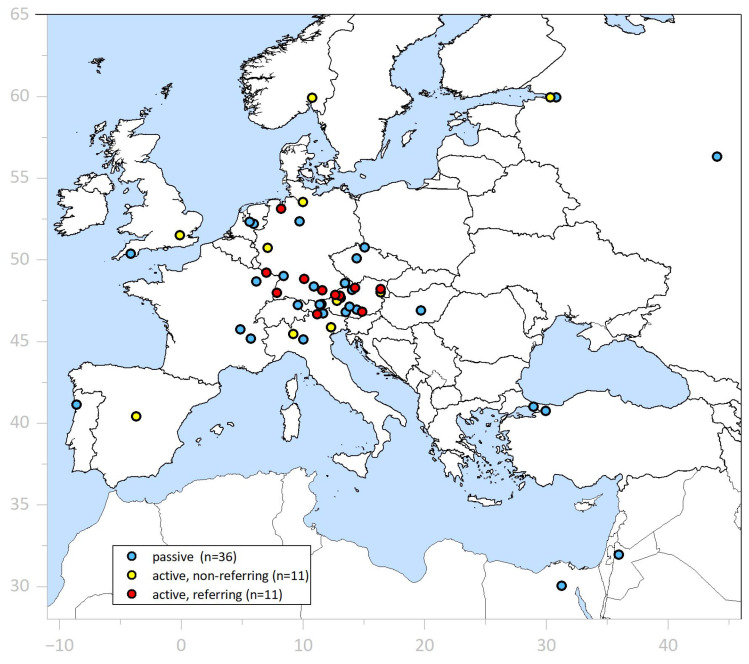
Distribution of reference centers with participating ESD-implementing endoscopists. Patient-referring active trainees (n = 11 (🔴)), non-referring active trainees (n = 11 (🟡)), and passive participants (n = 36 (🔵)) with clinical ESD experience from 15 countries, including Sydney/Australia and Wellington/New Zealand.

**ESD Tutoring Program** (Supplementary Section S1). ESD-implementing endoscopists from 22 endoscopic centers—11 referring their own patients and 11 non-referring—as well as 36 non-assisting ESD beginners participated (Figure 1).

All of them had been participating in an ESD Experimental Training & Update course [13] and had initial experience in ESD. Four internationally renowned Japanese ESD experts (N.Y., T.O., T.T., and Toshio Uraoka) performed complete ESD procedures (n = 28) in 16 patients during 5 ESD live events [13,14], in 7 patients during TUTORING events, and in 5 unpublished tutored cases in 2009–2010 (not included in [15,16]). The remaining 90 ESDs were performed by ESD beginners, with or without completion by tutors, in 20 tutoring sessions (each lasting 1–4 days, totaling 49 days). A contribution by the trainee of ≥25% of the total ESD duration was recorded in the register. ESD Clinical Tutoring is part of inpatient therapy and was offered free of charge to the trainees. We recommended endoscopy within ≤1 year and further follow-up (1–5 yrs) or additional therapy according to concurrent recommendations [17,18,19].

**Referral of patients**. Starting in November 2011, ESD-implementing centers of prospective trainees collected their cases for ESD tutoring at the University Hospital Salzburg. All 107 patients had been proposed for putative ESD with case vignettes, images, and the trainee’s optical diagnosis, and these data were forwarded to the Japanese experts. All 124 lesions fulfilled the criteria for endoscopic en bloc resection in Japan [17,19,20]. Early cancer lesions graded superficial–invasive were additionally investigated with high-resolution endoscopic ultrasound (hrEUS, 20 MHz) for sm-invasion criteria, and stage cN0 was confirmed with conventional EUS (7.5 MHz) or/and CT scan before embarking on ESD ([5] 1st ed. 2014). We excluded patients with evidence of lymph node or distant metastasis and—except for diagnostic ESD in the cardia and lower rectum—suspicion of deep submucosal invasion (≥sm2). As soon as the indication for ESD had been confirmed by the Japanese experts, informed consent was obtained, including the possibility of en bloc resection using the novel technique of ESD during tutoring and live events. In addition, the patient was specifically asked to consent in the tutoring, i.e., performance by the ESD trainee as well as the supervising Japanese expert, observation by a few ESD endoscopists for educational purposes, anonymized endoscopic video recording, follow-up of the patient’s outcome, and intent for publication (individual documentation of the above medical information provided to the patient was signed by both the patient and the organizer).

**Documentation for study and legal purposes.** In September 2009, the principal investigator instituted a prospective *register* (*no. 1*) of ESD-ITT in Excel and a document record file for the *untutored* ESD cohort [15,16], and in November 2009, an analogous *register* (*no. 2*) for ESD *involving external experts*. All ESD-ITT were documented in an Excel file for patient and lesion characteristics with visual classifications, endoscopic diagnosis, indications, operators, feasibility and safety parameters of ESD performance, such as duration (min) and dissection time by trainee, resection type (ESD, h-ESD, EID, pm-EMR), histopathological results, resection status (R1, Rx, R0, R curative) (Tables 2 in [9,10]), graded AE for inpatient procedures [21] (analogous AGREE grading is for *outpatient* procedures [22]), and follow-up for survival status, morbidity, and recurrent and metachronous NPL. The record file is based on hospital records, consents, anonymized full-length video recordings of each endoscopic procedure, ESD strategy protocols, CME feedback, and patient contact data. The CME program was in effect from November 2011 until March 2015. Complete long-term follow-up (Aug.–Dec. 2024) was obtained from hospital records, referring ESD physicians, and patients.

**Approval of ESD clinical tutoring.** According to §36 of the Austrian Medical Practitioners Act [23], the head of the department is entitled to apply for *teaching assistance* from international experts when implementing novel operations, such as ESD for early cancers and suitable precursor lesions. However, he/she bears the *medical and **legal*** responsibility for the endosurgical procedure on the patient. The requirements to approve Japanese experts as tutors and ESD-implementing gastroenterologists for assistance were met (with legal opinions issued in October 2011 by René Musey and Hans E Diemath, and confirmed in October 2024 by Johannes Zahrl, University of Vienna) (Supplementary Section S1). The implementation of *established* therapies and operations is not subject to ethics committee approval according to §30 of Salzburg County Hospitals Act (LGBI nr. 91/2010). Protocols no. 1, “*Implementation of ESD technique*”, and no. 2, “Clinical ESD Tutoring & ESD Live Events” (LEE), had been approved by the internal review board of the University Hospital (IRB protocol no. 93/02.04.2009; 510/03.11.2011). Protocol no. 2 was further accredited for continued medical education (CME) by the Austrian Medical Chamber in September 2011 and by the ESGE in May 2012 (on the recommendation of Meinhard Classen (†)).

### 2.2. Performance of ESD Procedures

**Optical diagnosis** was performed by the ESD beginners in their hospital to predict low-grade vs. malignant histology, and superficial vs. deep (sm ≥ 2) submucosa-invasion—using classifications and indications [9,24] detailed since 2009 in syllabus scripts (classifications updated through personal communication with T Oyama in January 2012 and N Yahagi in March 2012) and in the derived endoscopy atlas ([5] 1st ed. 2014) (for references see Supplementary Section S1).

**ESD techniques.** We applied CO_2_ insufflation and standard techniques for ESD with Dual-/J knife^®^, Hook-/J knife^®^ (Olympus Europe, Hamburg, Germany), Flush knife BT^®^, and Clutch cutter^®^ (Fujifilm Europe GmbH, Ratingen, Germany) [25,26,27,28] (see Supplementary Section S1). Specimen work-up and immunohistochemical/histopathologic evaluation in 2 mm serial sections were performed as previously described [9,15,16,29,30].

**Patients**. For the ESD Tutoring and LEE programs, 101 patients underwent ESD-ITT on 118 lesions. Among these patients, aged 68 years [37–91] and with an ASA score of 2 [1–4], 38% presented with co-morbidity ≥ grade 3, and 30% were on oral anticoagulant or/and antiplatelet drugs. For 91 (77%) ESD-ITT patients, the POSPOM score predicted increased postoperative mortality (POM) for alternative surgical resection (score 25–41, risk of POM 2.5–23%; Table 1A) [31]. The very elderly (83 ± 7.6 yrs; n = 12) with a score 34–41 (POM risk 15–25%) would have been very poor candidates for surgery on cancers in the esophagus, cardia, stomach, right colon, or large LST-GM in the hepatic flexure or anorectum—but tolerated curative ESD well. Most of these high-risk patients (n = 7) received antibiotic prophylaxis after uncomplicated, large-size ESD (d ≥ 5 cm) in the colorectum or stomach [32,33].

**Lesions.** Table 1B shows the organ distribution of all NPLs, including size (mv ± SD) and Paris classification. In 9 patients, 17 additional lesions were resected en block by ESD-ITT. Fifty percent of ESD-ITT were performed on advanced NPL (HGIEN or cancer), both by tutors and trainees. Retrospectively, all indications, except symptomatic esophageal SETs and duodenal adenomas, correspond to current guidelines [5,6,7,8,9,10,11].

**Table 1:** Characteristics of patients and lesions for ESD-ITT.

(A) Patient Characteristics													
		Details											
**Patients** (n, %)		101 (100%), 38% female/62% male											
**Age at ESD** (yrs)		68 [37–91]											
**ASA score** (1–5)		2 [1–4]											
**Co-morbidities** ≥ grade 3 ^1^ (38%)		CHF 26%, CVI 0.9%, CKD 5.6%, CPD 0.9%, LC 1.9%, IDDM 2.8%											
**Anticoagulation Therapy** ^2^ (30%)		OAC 12%, AP 16%, OAC & AP 0.9%, LMW heparin 0.9%											
**POSPOM score** (0–45) ^3^		27 [17–41] normal risk (POM < 2.5%) in 23% of 118 ESD-ITT											
**Increased POM risk, distribution** (77% of 118 ESD)		**Score**			**Risk** **(% POM)**		**All ESD (118)**			**Tutor ESD (28)**		**Trainee ESD (90)**	
		25–29			2.5–5%		**53%**			68%		**43%**	
		30–41			**6–23%**		**24%**			10.5%		**29%**	
**Intubation anesthesia**		1 hypopharynx-ESD, 17 esophagus-/cardia-ESD, 2 gastric ESD											
**(B) Lesions**													
	**Total**		**Esophagus**					**Stomach**	**Duoden.**		**Rectum**		**Colon**
			**SET**	**SC**		**Barrett**							
Patients (n)	101		5	8		9		10	8		22		39
ESD lesions ^4^ (n)	**118**		**5**	**8**		**9**		**14**	**9**		**24**		**49**
Malignant NPL ^5^ (n)	**59**		**0**	**8**		**9**		**8**	**1**		**16**		**17**
Size (cm) mean ± SD	4.6 ± 2.5		2.5 ± 0.9	5.1 ± 1.9		6.4 ± 2.6		4.4 ± 2.1	3.2 ± 1.9		6.9 ± 1.8		3.8 ± 1.5
0-IIb/LST-NG ^7^ (n)	39		-	5		1		2	-		1		30
0-IIa + c/LST-NGPD ^5^	23		-	-		1		11	1		2		8
0-IIa + Is/LST-GM ^6^ (n)	50		5 ^8^	2 ^9^		6 ^10^		1	4		21		10
0-IIa/LST-GH ^6^ (n)	7		-	1		1		-	4		-		1
Biopsy, targeted (%) ^11^	67%		40%	100%		100%		79%	56%		75%		43%

^1^ *Abbreviations*: CHF chronic heart failure (≥NYHA 3°), CVI history of cerebrovascular insult, CKD ≥ 3a chronic renal failure (eGFR ≤ 45 mL/min), CPD chronic pulmonary disease (arterial SaO2 < 90%), LC liver cirrhosis Child-Pugh B-C, IDDM insulin-dependent diabetes mellitus. ^2^ OAC oral anticoagulation (bridged with LMW heparin for ESD), AP antiplatelet therapy, LMW low molecular weight heparin. ^3^ POSPOM preoperative score to predict postoperative mortality (POM) [31]. ^4^ Indications conformed to ESD guidelines [5,9], except that ESD was recommended only for 5 symptomatic SET and duodenal adenomas [17]. ^5^ As verified by ER specimen histopathology. ^6^ ≥2.0 cm. ^7^ ≥3.5 cm. ^8^ Type 0-Is, hr-EUS benign criteria. ^9^ One type 0-Is in the proximal esophagus (→ diagnostic indication) and one type 0-IIb+Is lesion in the hypopharynx. ^10^ Including three type 0-Is lesions with irregular V/S structure (→ diagnostic indication). ^11^ Accuracy vs. ESD specimen: adenoma 95%, HGIEN 56%, carcinoma 77%.

**Figure 2:**
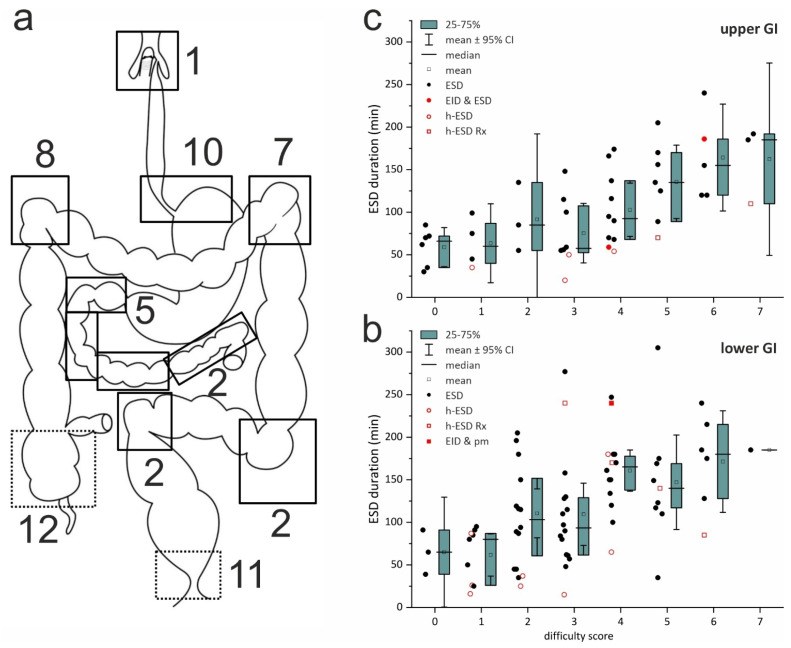
(**a**–**c**) Distribution of ESD-ITT in difficult sites and correlation of duration with difficulty score of ESD. (**a**) Anatomic sites with increased difficulty score (dotted line = +1 point, solid lines = +2 points) for the colorectum [34], and in exploratory modification for the upper GI tract. Numbers indicate the number of ESD-ITT procedures performed at these sites, accounting for a total of 50% of all ESD-ITT procedures. Graphs on the right panel show the duration (min) of ESD-ITT plotted against the ESD difficulty score of the lesion and location (**b**) for the validated score of Li in the colorectum and (c) for the score modified for the upper GI tract (compare Supplementary Figure S1), where the duration strongly correlates (R = 0.550, *p* < 0.00001) with the score as in the colorectum (R = 0.515, *p* < 0001). (**b**,**c**). Different ESD-ITT procedures; see icons: black filled circle = ESD en bloc, red open circle = h-ESD en bloc R0. In (**b**): red open square = h-ESD Rx [at score 3 due to endoscope with impaired angulation in the splenic flexure (Nov. 2009)]; red filled square = EID & pmEMR at score 4 for rectum LST-GM, see Supplementary Table S3, no. 6. In Figure 2c: red open square, duodenal h-ESD in 2 sessions at scores 5 & 7. Red filled circle = ESD & EID at score 6 for Barrett AC (Supplementary Table S3, no. 5), and STER & EID at score 4 for leiomyoma.

**ESD difficulty score** (**DS** 0–8; difficult ≥ 2) predicts the difficulty of a planned ESD procedure (Supplementary Section S1), is validated for correlation with the duration of ESD in the colorectum [34], and was modified for ESD in the upper GIT for the current analysis (Supplementary Figure S1). Retrospective DS analysis reveals that the majority of ESD lesions were difficult (DS 2–3; 35%) or very difficult (DS ≥ 4; 46%) (*secondary objective*, Figure 2a–c). Fifty percent of the lesions for ESD were located in the sites scored as most difficult (Figure 2a), and there were 14 of 16 recurrent lesions after EMR, ESD, or surgery scoring +2 DS points. The score best correlates with the duration of ESD-ITT, both in the colorectum (R 0.515, *p* < 0.0001)—confirming the validation—and in the upper GI tract (R 0.550, *p* < 0.00001), supporting its exploratory use (Figure 2b,c).

### 2.3. Data Analysis

**Study design.** The prospective cohort study has a modified ESD-ITT design, because we excluded 5 cases that the tutor assigned to alternative resection (pm-EMR, transanal microsurgery) and one published case [35] (Supplementary Table S1). The ESD-ITT dataset included 118 lesions in 101 patients. The *primary endpoint* was patient outcome (resection status and recurrence rate). *Secondary outcomes* were operability risk, trainee performance in optical classification and ESD indication (accuracy), and ESD performance on difficult lesions (feasibility and safety). Retrospective analysis of the anonymized data was granted by the Ethics Committee of Paracelsus Med. Univ. in March 2025 (2025_0005).

**Statistical analysis.** In the retrospective analysis, specimens’ area (as ellipsoid = ½ maximum length x ½ maximum width x π; in cm^2^), risk of surgical resection (POSPOM score), and the difficulty score (DS) of ESD lesions (Supplementary Figure S1) were calculated based on register no. 2 [31,34]. *Continuous parameters* were analyzed for mean ± standard deviation (SD), median [range], significance (*p* < 0.05) for difference with one-way ANOVA and Bonferroni’s adjustment, and for Pearson correlation. *Categorical parameters* were transformed into digit numbers—including endoscopic location (2 digits), classifications, difficulty score DS of ESD, and graded AE [21]—and tested with Spearman regression for significant correlation with continuous parameters, and with one-way ANOVA and Tukey post hoc test for significant differences (*p* < 0.05).

## 3. Results

### 3.1. Primary Objective: Outcome of All ESD Procedures

#### 3.1.1. Patient Outcomes

En bloc and curative resection rates were 95% (including 11% hybrid-ESD) and 88%, with no recurrence after R0 resection (Table 2A). En bloc rates were 98% for malignant and 92% (R0 88%) for benign NPLs, the latter due to elective hybrid-ESD (4%) involving final snaring in 2–3 fragments for four colonic and one duodenal adenoma (5 LGIEN, Rx; icons in Figure 2b,c). Hybrid-ESDs achieved clear margins and shorter procedure times. Supplementary Table S2A details the outcomes *by organ* for benign and advanced lesions.

#### 3.1.2. Non-Curative ESD and Recurrences

There were 5 recurrent lesions (4.2%): 3 cases after resection R1/Rx of adenomas (1 in the colon, 2 in the duodenum, all cured with a single hot snare polypectomy (HSP)), and an additional 2 cases after resection R1_h_/R1_v_ of 2 malignant lesions (Table 2A). The description of the final outcome (100% curative) after 7 non-curative ESDs (12%) is detailed in Supplementary Table S3, cases no. 1–7.

#### 3.1.3. Long-Term Outcome, Median Follow-Up of 9.8 [1.5–14.9] Years

As detailed in Table 2B, clinical follow-up was 98% complete for 59 patients aged 75 ± 11 years; 54 of them had endoscopic recurrence-free survival (RFS) of 9.1 ± 2.1 years. The other five patients with symptomatic esophageal SET stayed asymptomatic after ESD (4 granular cell tumors, R0)/submucosal tunneling resection (1 leiomyoma, R0) with normal endoscopic findings after 0.8 ± 0.3 years, and one suffered acute cardiac death at age 82 after 7.4 years. The 42 deceased patients died on average at old age after 5.7 ± 3.2 yrs (RFS 4.1 ± 3.0 yrs), one from *metachronous SCC* in the base of the tongue (OS 1.0, age 49 yrs), diagnosed in an advanced stage (cT4 N2b, M0) despite preceding UGI and ENT endoscopic follow-up. All other forty-one patients died from unrelated diseases, eight (19%) at age 75 ± 8 yrs from malignant diseases, and 33 (79%) from common causes at age 82 ± 8.5 years (73% males) matching normal life expectancy in Austria (males 78.5, females 83 yrs). **Metachronous colorectal lesions** occurred in only 11 of 73 patients (**15%**) during 10.3 ± 2.3 yrs. All were *adenomas* < 2.5 cm with/without LGIEN and were resected with EMR without any AE of DC grade > I (**FAP**, 2 cases; see Supplementary Section S2).

### 3.2. Secondary Objectives: Adverse Events and Outcome of Expert vs. Tutored ESDs

**Adverse events** (Table 2C). None of the 101 patients with 118 ESD-ITT had surgical repair or long-term morbidity (≥6 mo), and 30-day mortality was zero. AEs graded according to Dindo-Clavien (**DC**) occurred in **9.3%** [21]. A single AE (**0.8%**) was life-threatening (**DC IVa**) in an 85-year-old female patient with delayed perforation 1 h after ESD in the Billroth-II gastric stump and subsequent NSTEMI due to unexpected coronary artery disease, grade IV. AEs were moderately severe (**DC IIIa**) for 9 ESDs, i.e., transnasal endoscopy and antibiotics for *one aspiration* under propofol, endoscopic hemostasis for *5 delayed bleedings*, and repeated *endoscopic dilatations for stenosis* of the cardia (1 case, 3 times) or anorectal channel (3 cases, 3, 4, and 9 times). Of six inconsequential mini-perforations of the colorectum, all remained asymptomatic, with one receiving antibiotic prophylaxis after delayed clipping (**1 DC II**) and five after immediate clipping (**5 DC grade 0**).

### 3.3. Outcome of Expert vs. Tutored ESDs

#### 3.3.1. Diagnostic Performance

**Figure 3:**
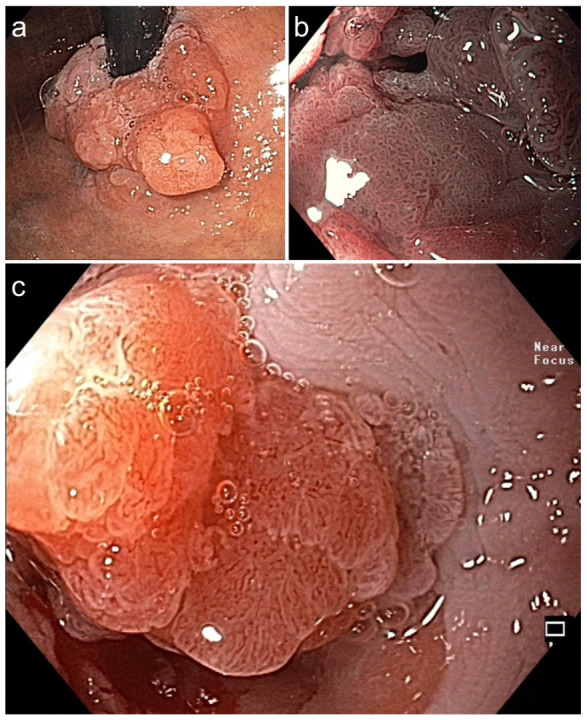
**Symptomatic circular Barrett adenocarcinoma 0-IIa+Is** for ***diagnostic ESD*** en bloc (16.6 × 6 cm). (**a**) in the cardia (WLI, retroflex view) and (**b**) in stenosis (BLI, 40×, prograde: dense VP & SP). (**c**) Dense SP (regular epithelial white zone) & VP with V/S concordance (WLI 60×). Histopathology: AC, G1 pT1a, m2 L0 V0 pN0 Bd1, ***R curative***. Died at age 87 from pneumonia, DFS 5.3 yrs.

**Figure 4:**
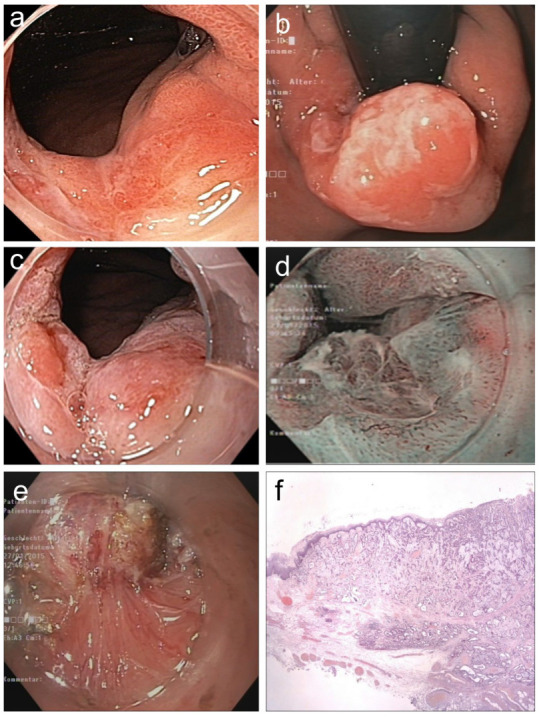
(**a**,**b**). **Barrett adenocarcinoma 0-IIa+Is, semicircular** (size 2.5 × 2 cm) in the cardia for ***diagnostic ESD*** (EUS uT1m uN0), standard WLI in prograde (**a**) and retroflex view (**b**). (**c**,**d**) A small area (0.5 cm) on the base of 0-Is with irregular SP and V/S discordance ((**c**) WLI 60×, (**d**) NBI, 60×). (**e**) pm-muscle retracting (**MR**) sign [36]. (**f**) Section (HE stain 100×) of AC margin showing pm invasion and resection R1 (right lower end): **AC G2, pT2, R1**. (Supplementary Table S3, no. 5).

As shown in Table 3A, trainees correctly staged 104 neoplasms on *optical classifications* (**OC**), as confirmed in the ESD specimens (***accuracy 88%***), ***over-staged 4%*** (4 LGIEN in LST-NG/PD as OC-superficial malignant and 1 rectum AC pT1a as VG-sm1), and ***under-staged 8%*** (6 HGIEN as OC-benign in LST-GM, 1 SCC 0-Is pT1b, sm2 as OC-sm1, and 2 AC pT2 in cardia and lower rectum as OC-sm ≥ 2). Endoscopic grading yielded ***89% curative indications*** for ESD of 105 NPL (104 *benign* + superficial-malignant with *classical criteria* plus *1 NET* cN0). Among 13 carcinomas cN0 graded sm-invasive, six (***5%***) had ***curative* ESD indications** for ***expanded criteria***, and seven (***6%***) had ***diagnostic indications***—one for malignant cardia stenosis (Figure 3a–c) and five for likely *deep invasion* into submucosa ≥ sm2, i.e., 1 esophagus SCC 0-Is (79% risk of sm ≥ 2 invasion [5]), 3 Barrett AC 0-Isp/0-Is (Figure 4a–f), 1 lower rectum LST-GN (size 13 × 7 cm) with a cancer nodule—and 1 anastomotic recurrent SCC 0-IIb for *high risk* of resection *R1_h_* without safety margin at the anastomosis. For *muscle retracting signs*, the expert converted ESD on both deep-invasive AC to intermuscular dissection (**EID**) in the cardia (Figure 4e) and in the lower rectum [36] enabling curative surgical en bloc resection (for AC pT2; R1, see Supplementary Table S3, nos. 5; 6).

#### 3.3.2. ESD Performance

Trainees started as beginners at the initial learning or early competence level (2–42 prior ESDs) (see Table 3B). When comparing the ESD difficulty score (DS) of lesions, referring trainees were involved in a similar proportion (29%) of very difficult lesions (DS ≥ 4) as sole tutors (32%), and non-referring trainees were involved in a proportion three times lower. Conversely, all trainees performed ESD on lesions with 33% smaller areas and twice as often on easy lesions (DS ≤ 1) compared with sole tutors. The differences between the groups are non-significant, and the performance levels of trainees reflect the quality of expert supervision and tutoring.

Nevertheless, trainees achieved self-completion of a median **75%** [25–100%] of ESD-ITT, with high rates of en bloc (**96%**) and R0 resection (**94%**) that were similar in both subgroups—proving effective instructions and supervision during the procedures. Tutors performed ESD with professional average speed (9.3 ± 6.2 cm^2^/h, *p* = 0.12, Tukey (ANOVA) compared to trainees’ group), including the largest and most difficult lesions (22 ± 22.5 cm^2^; DS 4 [0–7]). Tutors furthermore ensured that average duration (112 ± 59 min) of trainee ESDs did not exceed that of tutor ESDs (122 ± 69 min; *p* = 0.47, Tukey (ANOVA)), and average performance speed (7.5 ± 5.2 cm^2^/h) was maintained on a skilled level—by completing slower or very challenging procedures. Most CME procedures (95%) were completed within 3 h during the routine program. ESD trainees were involved in demanding ESDs and rated the tutoring as excellent CME, which was very helpful for ESD in their practice (Supplementary Figure S2).

## 4. Discussion

Clinical tutoring by experts has rapidly implemented a network of professional centers for the novel, groundbreaking ESD technique in Japan [3,4]. This study aimed to transfer the ESD technique from Japan to the West with ESD Clinical Tutoring based on teaching assistance by a Japanese expert during routine endoscopic treatment. The main findings of our analysis are that (i) patient outcomes meet professional standards for ESD as validated in Japan [7,8,9,11] and (ii) trainees achieved high rates of correct optical diagnosis as well as good clinical efficacy of ESD procedures.

The **data** demonstrate an 88% curative resection rate for malignant lesions and zero recurrence after R0 resection of benign lesions in the setting of tutored ESDs. These data are far better than those reported for the untutored implementation of ESD. Thus, a meta-analysis of western ESD series (mainly during the stage of untutored implementation) reported mean en bloc resection rates of 81%, R0 rates of 71%, and overall curative rate of 67% [3]. Analyses on simultaneous learning curves (up to 80–120 untutored ESDs) reported en bloc, R0, and curative resection rates of 75–85%, 65–74%, and 56–65%, respectively [16,37,38,39,40,41,42]. All our patients remained recurrence-free after endoscopic resection of 3 recurrent adenomas (at R1/Rx) and proper management of 7 non-curative ESDs. In addition, our trainees achieved a high rate of accurate optical diagnosis (88%) and ESD indication (96%) for benign and malignant lesions. ESD trainees under-staged two cases of deep cancer infiltration. In these cases, the expert applied endoscopic intermuscular dissection, which could accurately diagnose a pT2 G2 adenocarcinoma in the cardia and lower rectum, respectively, and subsequently enabled curative surgical en bloc resection. Tutored ESD trainees achieved regular procedure times (1–3 h) and maintained a skilled resection speed even for very difficult lesions. In addition, trainee self-completion rate was high (median 75; 25–100%), which is comparable to the self-completion rate of 74% that was achieved by skilled Japanese trainees during their first 40 tutored colorectal ESDs [2]. On the one hand, there were trainee ESDs with 100% self-completion even on challenging complex lesions (DS 4–7) without recurrences. On the other hand, tutors managed that the duration of trainee ESD did not exceed that of tutors. The en bloc resection rate of 95% included 11% hybrid-ESD en bloc to shorten the duration of ESD, e.g., in the case of 2 or 3 colonic ESD in a single session. Elective final snaring with low current power showed precise margins of specimen and clean resection bed without any AE—confirming the findings of the original publication [27]. In contrast, *conversion to hybrid*-ESD during *untutored* ESD-ITT mainly serves for self-completion in cases of long duration or perforation [15,16,37,38,39,40,41], which likely explains poor en bloc (68%) and R0 (61%) rates, as well as high perforation rate (5%) [3].

Finally, the safety profile of the procedures performed during the tutoring program was favorable, which is remarkable since many elderly patients had a predicted high postoperative mortality rate. Thus, the outcome of the tutoring program meets the requirements for routine clinical therapy.

**Limitations.** While this study is unique, we do acknowledge several limitations: (i) The modified study design retrospectively analyzed novel predictive scores for the risk of alternative postoperative mortality and ESD difficulty of the lesions. However, based on a complete record file, this additional characterization provides an exemplary register for prospective studies on endoscopic resection of complex and putative malignant neoplasms. POSPOM score anticipates risk for surgical en bloc resection [31], while ESD en bloc is becoming an alternative for surgery in oncological regimens [43]. The predictive ESD difficulty score—pending validation for the upper GI tract—may improve strategy for and outcome of difficult ESD, and provide cut-off values for operator skills as well as referral to expert centers [44]. (ii) The indications were prevalence-based and thus included a high proportion of colorectal and esophageal lesions. However, since early gastric cancer—the typical beginner’s lesion in Japan—is less prevalent in Europe, this approach reflects the clinical reality. (iii) The lesions referred were biased towards difficult and challenging ESD—yet the steep gradient of ESD difficulty highlights the quality of our tutorials and was reflected by positive feedback from the trainees—many of whom subsequently published their own ESD data [13,15,16,39,41,45,46,47]. (iv) Finally, the study was conducted at a single center with Japanese experts and trainees with initial ESD experience and therefore might not be fully generalizable to ESD proctoring in a Western setting, even though ESD expert centers have been recently established in Europe.

Indeed, it has to be emphasized that the success of the ESD clinical tutoring concept only applies for *strict preconditions*: the *Japanese top expert tutors* with profound ESD teaching and tutoring experience, interventional endoscopists nearly on ESD competence level from major centers, referring patients with NPL classified for indication and difficulty of ESD to a single center, and medical and legal responsibility of the head of department for the ESD involving referring doctor and tutor. Nevertheless, such tutoring experts with refined ESD techniques [43,44,48], shall be available for another decade to advance professional ESD performance. This ESD Clinical Tutoring concept—with the co-operation of 6–10 centers—could serve to establish networks of Early Cancer Centers (e.g., 1 per 5–7 Mio. population) in Europe [9].

### Implications of This Study

This Clinical Tutoring concept obviates the exclusion of very frequent colonic indications—and other challenging lesions—from the stage of ESD implementation. Furthermore, some western endoscopists successfully implemented colorectal ESD in an untutored fashion with technical step-up and good safety [15,16,38,39,40,41,42], and one later introduced ESD on early cancers of the upper GI tract with near professional performance level [46].

In addition, Tutoring ESD sessions included the most difficult lesions for ESD and may have reduced the need for alternative oncological surgery, as well as enhanced the learning curve of ESD beginners, thus saving resources and costs.

Between 2011 and 2015, this was the only one-to-one hands-on clinical training course in Europe for both ESD indication and electrosurgical ESD skills, led by top experts from Japan. This training course proved feasible for routine endoscopic treatment. At that time, this represented the best minimally invasive treatment for early GI cancer patients available in Europe. The ESD Tutoring continued as an ESD Clinical Tutoring Course in Munich 2019 (Organizers: Ch Schlag, R Schmid & F Berr) (further on interrupted by COVID-19 pandemia), and 2016–2025 as CME events in Freiburg (Organizer: H-P Allgaier, I Steinbrück), Bonn (Organizer: F-L Dumoulin), Germany, and as annual eNNdo Days in Nizhniy Novgorod (Organizer: A T Mitrakov), Russian Federation.

## 5. Conclusions

ESD Clinical Tutoring Courses under Japanese expert supervision ensure excellent patient outcomes and consistent procedural standards during routine treatment. This model may foster professional ESD performance across European referral centers and—as a cooperative program—promote networks of Early GI cancer Centers in Europe.

## Figures and Tables

**Table 2:** Patient outcomes of ESD-ITT.

(A) Outcome of ESD (%)						
	All NPLs		Advanced		Benign	
n	118		59		59	
En bloc	**95%**		98%		92%	
R0	91%		94%		**88%**	
R curative	**88%**		**88%**		-	
R1/Rx	**7.6%**		6.8%		8.5%	
Recurrence at R1/Rx	**4.2%**		**3.4%**		**5.1%**	
2nd ESD ^1^/ER of recurrence ^2^			**1.7%** ^1^		5.1% ^2^	
R non-curative	-		**11.9%** ^3^		-	
Oncosurgery curative			**6.8%**			
Long-term DFS/RFS	**100%**		100% ^1,3^		100% ^2^	
**(B) Long-term outcome**						
**Survival Status**	**Patients** **n**		**Follow-Up ^4^** **yrs**	**Age at End** **yrs**	**OS** **yrs**	**RFS ^5^** **yrs**
**Alive**	**59**		11.2 ± 1.6 ^4^	75 ± 11	-	9.1 ± 2.1
**Deceased**	**42** (67% m)		5.7 ± 3.2	80 ± 10	-	4.1 ± 3.0
**Causes of death**						
**Recurrent disease**	**none**					
**Metachronous SCC** ^6^	**1**		2.7	**49**	1.0	2.3
**Non-related malignant** ^7^	**8**		7.5 ± 3.9	**75 ± 8**	2.9 ± 2.3	6.8 ± 3.2
**Non-malignant diseases** ^8^	**33**		5.4 ± 2.9	**82 ± 8.5**	**-**	3.8 ± 2.6
**(C) Adverse Events**						
	**All ESD (n)**		**Dindo-Clavien Grade** [21]			
	**%**	**118**	**IVa**	**IIIa ^9^**	**II**	**0**
Perforation		2	1 ^10^	-	1	(5)
Aspiration (under propofol)		1		1 ^11^		
Delayed bleeding		4		5 ^12^		
Stenosis (cardia/anorectum)		4		4		
Total AE	9.3%	11	0.8%	7.6%	0.8%	**-**

Table 2A: ^1^ curative ESD of recur. colon AC pT1b. sm1 (27 mo after ESD of HGIEN, R1); ^2^ single HSP ± APC without recurrence; ^3^ 1 eso-SCC pT1b, sm2 had adjuvant radiotherapy; 1 Barrett AC G2 pT1b, sm2 only follow-up (DFS 9.5 yrs), one 2nd ESD, 4 curative onco-surgery: all 7 achieved long-term DFS (see Supplementary Table S3). Footnotes, Table 2B: ^4^ Clinical follow-up 98% complete with 2 lost (LGIEN, R0) after 6/5 yrs follow-up without recurrence at age 43/82 yrs. ^5^ Endoscopic follow-up is 97% complete: 3 heart patients with negative FOBT on OAC refused colonoscopy after curative ESD (OS 2.1, 3.6, 2.9 yrs, died at age 67, 91, 94 yrs); 5 SET only had endoscopic 1 yr follow-up. ^6^ In the base of the tongue diagnosed 1.7 yrs after curative ESD of esophageal SCC. ^7^ Progressive disease of hypopharyngeal SCC, carcinomas of the bile duct, prostate, pancreas, lung, CMML, follicular lymphoma. ^8^ 61% cardiovascular, 15% pulmonary, 9% cerebrovascular, 6% thromboembolic and 9% infectious. Abbreviations: FOBT fecal occult blood test, OAC oral anticoagulants, OS overall survival. Footnotes, Table 2C: ^9^ Managed with endoscopic therapy (broncho-gastroscopy; hemostasis; dilatations). ^10^ 1 h after curative ESD in Billroth-II gastric remnant due to a small serosa patch exposed by the tutor during ESD → perforation closed with 2-row clipping [36], antibiotics → leakage at 1 clip → short bacteremia → NSTEMI that was cured with a stent into the main LCA and afferent-loop drainage to collect biliary–pancreatic secretion for 17 d until discharged home. ^11^ 30 min interruption of anorectal ESD. ^12^ 1 episode of bleeding in cardia stenosis. Abbreviations: LCA left coronary artery, NSTEMI non-ST-elevation myocardial infarction.

**Table 3:** Trainees: Optical classification and ESD performance.

(A) Optical Classification (OC)					
		OC Diagnosis	OC Correct	Histopathology	n
Benign		61		Benign 50%	59
• SET		5	5	• SET	5 ^1^
• adenoma		56	52	• LGIEN	54
Malignant		57		Malignant 50%	59
• non-invasive		43	37	• HGIEN/pT1a	46
• sm1		9	7	• pT1b sm1	7
• ≥sm2		4	2	• pT1b ≥ sm2	3
pT2		-		pT2	2 ^2^
• NETsm3uT1 N0 ^3^		1	1	• NEC G2 pT1 ^4^	1
Total NPL		118	104		118
**OC accuracy**			**88%**		
**(B) ESD Performance**					
		**Tutors Only**	**All Trainees**	**Referring Trainees**	**Non-Referring Trainees**
n		5	22	11	11
Prior ESD experience (n)		n. g.	14 [2–42]	15 [5–42]	11 [2–20]
Participation, Tutoring + LEE (d)		15 [3–30]	15 [5–54]	17 [7–54]	13 [5–49]
ESDs (n)		**28** ^5^	**90** ^6^	**57** ^6^	**33** ^6^
Malignant NPL		14	45	30	15
ESDs per endoscopist		4 [1–14]	3 [1–12]	3 [1–12]	2 [1–7]
ESD difficulty score (DS, x ± sd)		**3.8** ± 1.9	3.1 ± 1.9	**3.4** ± 1.8	**2.6** ± 1.7
Easy DS 0–1		**11%**	22%	**19%**	**27%**
Very difficult DS ≥ 4		**32%**	24%	**29%**	**12%**
Self-completion (%)		100	**75** [25–100]	**68** [25–100]	**75** [25–100]
Duration (min)		**121** ± 69	**112** ± 59	**119** ± 61	100 ± 56
Maximum diameter (cm)		5.4 ± 3.2	4.4 ± 2.2	4.6 ± 2.1	4.1 ± 2.4
Area (cm^2^)		**21.9** ± 22.5	**14.7** ± 17.2	14.7 ± 12.0	14.6 ± 23.9
Speed (cm^2^/h)		**9.3** ± 6.0	**7.5** ± 5.2	7.4 ± 4.4	7.6 ± 6.6
Resections (%)	En bloc	93%	96%	95%	97%
	R0	82% ^7^	94%	91%	97%
	R curative	79% ^8^	96%	87%	100%

^1^ 4 granular cell tumors (ESD) and 1 leiomyoma (submucosal tunneling resection and EID, R0). ^2^ Both NPLs without a break of sm-band on hr-EUS/EUS for diagnostic ESD of Barrett-AC in the cardia and LST-GN with sm-invasive cancer nodule in the lower rectum. ^3^ SET (15 × 12 mm) visualized on a somatostatin receptor scinti scan. ^4^ NEC G2 pT1 L0 V0; R0. ^5^ ESDs 100% self-performed by 4 Japanese experts (Tutoring program) and 1 German expert (LEE only). ^6^ ESDs (n) ≥ 25% self-performed by trainees with or without completion by tutors. ^7^ *Due to **3 ESDs*** Rx/R1 of adenomas with ***improper endoscopes*** in 2009/2010 and ***2 diagnostic*** ESDs R1 (Supplementary Table S3 no. 1, 6). ^8^ *Due to **3 diagnostic***, ***non-curative*** ESDs (Supplementary Table S3 no. 1, 4, 6).

## Data Availability

All relevant data are included in detail in the manuscript and Supplementary Materials.

## Supplementary Materials

The following supporting information can be downloaded at https://www.mdpi.com/article/10.3390/jcm15020675/s1: Section S1. Supplementary Approval [49] Supplementary Methods: Optical classifications [50,51,52,53,54,55,56,57,58,59], endoscopic en-bloc resection techniques [60,61,62,63,64,65]. Section S2. Supplementary Results including Supplementary Table S1 Excluded cases scheduled for ESD Tutoring and LEE Program, Supplementary Table S2A Detailed Outcome of ESD-intention to treat (ESD-ITT), Supplementary Table S3 Outcome of non-curative ESD-ITT, Supplementary Figure S1 Predictive ESD Difficulty Score for lesion items and anatomical location, Supplementary Figure S2 Feedback of ESD implementing endoscopists for the ESD Tutoring program.

## Abbreviations

The following abbreviations are used in this manuscript:

AC	adenocarcinoma
AE	adverse event
APC	argon plasma coagulation
BLI/NBI/WLI	Blue-/Narrow-Band-/White-Light-Imaging
CME	Continued Medical Education
ESD	Endoscopic Submucosal Dissection
h-ESD	hybrid-ESD
-ITT	-intention-to-treat
ESGE	European Society of Gastrointestinal Endoscopy
FAP	familial adenomatous polyposis
G1/G2/G3	grading G1/G2/G3
GI(T)	gastrointestinal (tract)
JNET	Japan NBI Expert Team
HGIEN	high-grade intraepithelial neoplasia
HSP	hot snare polypectomy
LGIEN	low-grade intraepithelial neoplasia
NET	neuroendocrine tumor
NEC	neuroendocrine carcinoma
NPL	neoplasia
pm	proper muscle layer
pmEMR	peace meal Endoscopic Mucosal Resection
mNBI	magnifying NBI
sm	submucosa(l) layer
OC	optical classification of NPL
OS	overall survival time
DFS	disease-free survival time
RFS	recurrence-free survival time (for benign neoplasias)
SCC	squamous cell carcinoma
R1h/R1v	resection with positive horizontal/vertical margin
SET	subepithelial tumor
