# Supplementary material for: Clinical Endoscopic Submucosal Dissection of Trainees Tutored by Experts—ESGE Endorsed Courses and Live Endoscopic Events 2011–2015
Source: J Clin Med. 2026 Jan 14;15(2):675. doi: 10.3390/jcm15020675 (PMC12841996; doi:10.3390/jcm15020675)
Supplement: Supplementary file 1 [file jcm-15-00675-s001.zip › jcm-4037041-supplementary.pdf]

## Supplement

## Section S1. Methods

**ESD Tutoring Program** was part of inpatient therapy and a CME program for ESD implementation [1]. One of the ESD endoscopists assisted the expert, and between 4 and 6 ESD endoscopists observed the procedure close by in the endoscopy room with an additional TV screen linked to the endo-tower. The referring ESD endoscopist was present while settling the patient for ESD, and the passive participants joined when the patient was under anesthesia. *Non-referring* ESD endoscopists were involved as ESD assistants on the lesions (n= 69) referred *from Salzburg county*. The ESD assistant physician in presence of the organizer demonstrated extension and classifications of the lesion, and proposed the indication and strategy for ESD. Then the Japanese expert took over the scope, checked the accurateness of extension and optical classification of NPL, and of indication and strategy for ESD, and performed or supervised marking the safety margin. Less experienced ESD physicians were handed over the scope for short intervals to perform typical maneuvers of dissection during stable scope position. When skilled enough, the assisting physician was allowed to continue as long as the progress was adequate. Participants received a hand-out of anonymized case vignettes and endoscopic images, and selected tables (classifications, ESD techniques & power settings) of the ESD syllabus script (on www.early-cancer.eu). After 2 weeks they received an email with vignettes of resection procedure, outcome, and few highlight endoscopic and microscopic images. An atlas (containing 47% on images from this CME program) provided endoscopic differential indications (1st ed. May 2014 [2]). The organizational costs of the ESD Tutoring program were relatively low, and covered by a foundation[1], because we added the events to scheduled visits of the tutor to Europe.

**Approval of ESD Tutoring**. *Teaching assistance* is legalized in most European States as in Austria [3]. The patient has a contract with the hospital for diagnostics and treatment by the medical team led by the head of department. According to §36 of the Austrian Medical Practitioners Act [3], the head of the Department is entitled to apply teaching assistance by international experts when implementing novel operations – such as **Clinical Tutoring** for ESD - when he/she bears *medical and legal responsibility* for the endosurgical procedure in the patient. This differs from **Proctoring**, when the trainee bears the legal responsibility performing the operation under instruction by the expert, and **Preceptoring**, when the trainee performs the operation under legal responsibility of the expert. The hospital has insured the head of department against any claims by patients from diagnostic and interventional procedures, except in cases of intent. The European States mutually recognize Medical Board Licenses for gastroenterologists. ESD for proven indication is only legalized as hospital inpatient procedure. The patient has the right to freely choose the physician in European States, and the costs

[1] Sponsor see Acknowledgements.

for inpatient treatment will be reimbursed to the extent that they would be incurred in the home country, when equally effective treatment is not available in regional hospitals [4]. ESD of early malignant or complex mucosal GI neoplasias had been validated in Japan as superior endoscopic operation that benefits the patients [1], and ESD equipment is certified. Thus, the requirements are met to approve experts from Japan as tutors and European ESD-implementing endoscopists as assisting physician for ESD CME[2].

**Endoscopy and optical classification (OC).** For chromoendoscopy of lesions we used Lugol solution (right before ESD) in the esophagus, acetic acid with/out indigo carmine in Barrett´s esophagus and stomach, and indigo carmine - or targeted crystal violet for enhancement of Kudo pattern $V_I$ - in the colon. We focused on Paris classification plus macroscopic signs (+/- air-induced deformation, alterations of folds, distortion of mucosal areal structure) [5], and in magnified (60-120-fold) WLI and NBI/BLI imaging on irregular patterns with demarcation line, for capillary loops in esophagus [6,7] (since 01/2012)[3], mucosal surface (S) and vascular (V) patterns and V / S con-/discordance in stomach [8,9][3], Barrett esophagus [10,11][3], colorectum [12-14][4], and on findings in remission of ulcerative colitis [15] as detailed ([2], 1st ed. May 2014). Single targeted biopsy had been taken in the majority of lesions (67%) to ensure the diagnosis of neoplasia for the patient. The accuracy of biopsy vs. ESD specimen was 94.7% for adenoma +/- LGIEN, 56% for HGIEN, and 77 % for cancer. Optical diagnosis followed the classifications and indications detailed in annual syllabus scripts (since 2009) [3,4] and the derived endoscopy atlas (1st ed. May 2014 [2]), and summarized from guidelines (Tab. 1 in [1]).

Initial equipment was gastroscope GIF-T160® OLYMPUS without water jet, and EG530D®, FUJIFILM with water jet and upwards angulation 210° - poor maneuverability for difficult lesions (DS 3 – 4) caused 3 hybrid-ESD of long duration (**Fig 2b,** see icons). From Jan. 2012 we used endoscopes with improved angulations and focused water jet (GIF-H180J, CF-H180J; ESD no. 8 – 64; CF-H180AL, PCF-H180-I, ESD no. 65 - 112), and from June 2015 advanced magnifying ESD endoscopes (GIF-HQ190J, CF-HQ190J, PCF-H180AL, OLYMPUS, ESD no. 114 - 119) – and simultaneously equivalent FUJIFILM endoscopes from June 2013 -2018.

**ESD technique** was performed with Dual Knife®/-J® and/or Hook Knife®/-J® (OLYMPUS), Flush Knife BT® or Clutch Cutter® (FUJIFILM), straight- or conical-shaped hoods (Olympus; Fujifilm), and for large vessels Coagrasper® hemostatic forceps (OLYMPUS) powered by VIO-300D® or VIO-3® generator (ERBE Medizintechnik, Tübingen, Germany) [16-19]. Depending on the lesion and tutor´s preference, we applied the partial- (pci-) or initial complete (icci-) circumferential incision- or tunneling-methods for ESD using gravity and tissue retraction forces or the traction-line (in esophagus, cardia, and rarely left colon) [20-23], submucosal tunneling endoscopic

[2] According to expertise of René Musey for medical and insurance law, and Hans E Diemath for medical law / Salzburg in Oct. 2011; confirmed with expertise by Johannes Zahrl, head of Section Medical Law, Univ. Vienna / Vienna in Oct. 2024.
[3] updated by T Oyama, personal communication 01/2012
[4] updated by N Yahagi, personal communication 03/2012

intermuscular dissection (STER & EID) for leiomyoma, and EID during diagnostic ESD of deep-invasive carcinoma in cardia and rectum [16,24]. We rarely applied hybrid-ESD with monofil snare (OLYMPUS) and *low* power (dry cut 30 - 40 W) for en-bloc resection (n=12; 11%), and 5 times for final resection of the specimen in 2 – 3 pieces after widespread circular submucosal dissection (**h-ESD**, Rx) [25]. We used for gastroduodenal ESD a flexible overtube (US Endoscopy, Vienna). Twenty ESDs were under intubation anesthesia, the remaining 99 ESD sessions under intravenous anesthesia with midazolam and propofol. **Histologic assessment** of was based on the Vienna classification and WHO criteria [26-28]. The Image-documented, pinned ESD specimens were fixed in 4% buffered formaldehyde solution. Serial microscopic examinations of 2 mm thick sections of the whole specimen were characterized for neoplasia, depth of any submucosal invasion, lymphatic, vascular or perineural invasion, and completeness of resection (R0, R1, Rx) as described [26,29].

**Lesions for difficult ESD-ITT.** The high percentage of lesions in colorectum and esophagus reflects regional *prevalence of indications*. ESD-implementing participants performed untutored ESD on the least difficult lesions in their hospitals, and referred their most difficult lesions for ESD TUTORING (*referral bias* towards very difficult ESD) – as shown in retrospective analysis of **ESD difficulty score**. The degree of difficulty of an ESD procedure was scored for colorectal lesions according to Li B et al[30], that correlated most significantly (R 0.515, P< 0.0001) with *duration time* of the colorectal ESD-ITT confirming the validity of the score [30]. In exploratory fashion, we extended the principles of the difficulty score for ESD-ITT to the upper GI tract. We used the same scorings for tumor size, circumferential extension, tumor morphology, and we defined unfavorable anatomical locations including in Billroth-II gastric stump (**Suppl. Fig. 1**).

**Study design - Excluded patients** (n=6; **Suppl. Tab. 1**). One scheduled male patient was referred for another transanal endoscopic microsurgery (TEM: LGIN, R0; RFS 5.2, OS 9.7 years, acute cardiac death at age 89 yrs). One LEE-ESD of a giant lesion (17x10 cm) with endoscopic suturing for rectal reconstruction has been published [31]; this male patient was discharged after 2 days and remained without AE nor recurrence (age 59 yrs; RFS 6.7 yrs). For **4 duodenal adenoma** patients, the tutor a priori preferred complete resection with **pm-EMR**.

## Section S2. Results

**Suppl. Tab. 2A** reports the *short-term outcome* of ESD-ITT detailed for the ***organ distribution*** of benign and advanced NPL.

**Suppl. Tab. 3 Non-curative ESD** details the outcome of these seven cases (**12%** of malignant lesions):

Case no. 1 had diagnostic ESD of recurrent anastomotic SCC after distal esophagectomy and recurrence again after 10 mo (cured with proximal esophagectomy). No. 7 showed recurrence at delayed 1st control 2.3 years following ESD of colon LST-NG (HGIEN; $R1_v$), and returned for the 2nd ESD (curative) by his tutor. The other 5 had diagnostic ESD for graded deep submucosal invasion: No. 2 with SCC type 0-Is (ESD: pT1b sm2 **L1** → risk 30-50%) had adjuvant radiation therapy without recurrence. No. 3 with Barrett carcinoma 0-Is (ESD: pT1b sm2 [smi 729 µm] → risk 5 – 10%) only had follow-up. No. 4 with deep sm2 invasion [856 µm] of Barrett carcinoma underwent curative distal esophagectomy & proximal 1/3 gastrectomy (without residual disease). In cases no. 5 and 6, hr-EUS had supported indication for diagnostic ESD that required EID for pm-invasion (pT2 L1, R1). EID enabled curative oncologic surgery en-bloc. Overall, 4 patients (6.7%) underwent curative oncologic surgery en bloc.

**FAP metachronous adenomas.** Two colectomized female FAP patients had syn-/metachronous gastric adenomas (< 1.5 cm, one resected with en-bloc EMR, R0) as well as multiple duodenal adenomas that were down-staged to Spigelman score I with cold snare polypectomies (all LGIEN) every 3 years [2] – one was 80 years old at time of follow-up (12 years), the other died from cholangiocarcinoma (OS 1, age 75, follow-up 11 years).

**CME format of ESD Tutoring**. In all ESD TUTORING events 2011 - 2015, participants have rated the tutor´s teaching excellent, the optical grading for accurate differential indication and the instructions for high skills very helpful to progress to skilled / near- professional level of untutored ESD in their home hospitals. Feedback of participants was rating between excellent and good on a scale from 1 to 5 (**Suppl. Fig. 2**).

## Supplementary Tables

### Suppl. Table. S1 Excluded cases scheduled for ESD Tutoring & LEE Program

<table>
<tr><td rowspan="3">Patients excluded from scheduled ESD-ITT (124 neoplasias, 107 patients)</td><td colspan="5">1 ESD abandoned for recurrent adenoma after TEM (LGIEN 25x18mm, severe sm-fibrosis) → $2^{nd}$ TEM-OP, no recurrence (5 yr), cardiac death after 9.7 yrs.</td></tr>
<tr><td colspan="5">1 ESD published [31]: large size 17 x 10 cm & long duration for ESD (LGIEN & HGIEN, R0) & rectal reconstruction with endo-suturing<br>→ No AE (discharged on day 3), no recurrence (59 yrs; RFS 6.5 yrs)</td></tr>
<tr><td colspan="5">ESD-ITT for 118 neoplasias in 101 patients → see Tab. 2</td></tr>
<tr><td rowspan="5">Patients with duodenal adenoma for pmEMR-ITT</td><td colspan="5">3 [1] complete pm-EMR, LGIEN, Rx; mucosal closure. Median 50 [30-50] mm, duration 44 ± 7 min → No AE, Dindo-Clavien grade 0</td></tr>
<tr><td colspan="5">1 pm-EMR of recurrent adenoma+LGIEN in pars 4 (60mm, circumferential), duration 240min → 1 AE, Dindo-Clavien grade IIIa[2]</td></tr>
<tr><td>Follow-up (n)</td><td>interval (yrs)</td><td>age (yrs)</td><td>ASA score</td><td>RFS (yrs)</td></tr>
<tr><td>4</td><td>11 ± 1.5</td><td>79 ± 2.4</td><td>all I or II</td><td>n=3: 8.6 ± 1.0</td></tr>
<tr><td colspan="5">2 (50%) recurrent cases<ul><li>1 small LGIEN after 1.5 yrs, no recurrence after HSP&APC, RFS 7.6 yrs</li><li>1 small LGIEN every 2 yrs (→ HSP & APC), age 81 yr, ASA II, RFS 2 yrs → satisfactory outcome (without resective surgery).</li></ul>No AE ≥I grade, Dindo-Clavien for all HSP&APC in both patients</td></tr>
<tr><td colspan="6">[1] 1 pm-EMR performed by referring ESD endoscopist, the other 3 by the tutor. [2] small retroperitoneal perforation and recurrent bleeding → coiling of arteria duodenalis, 6 units blood, antibiotics.<br><u>Abbreviations</u>: AE adverse events, APC argon plasma coagulation, HSP hot snare polypectomy, TEM transanal endoscopic microsurgery.</td></tr>
</table>

## Suppl. Table. S2A Detailed Outcome of ESD-intention to treat (ESD-ITT)

| **(A) Short-term outcome** | **% Rate** | **Esophageal NPL** | | | **Gastr. NPL** | **Duoden. NPL** | **Colon NPL** | **Rectum NPL** |
|---|---|---|---|---|---|---|---|---|
| | | **SET** | **SCC** | **Barrett** | | | | |
| **Benign NPL** (n = 59) | | 5 | - | - | 6 | 8 | 32 | 8 |
| **En bloc** (%) | **92%** | ≡ | - | - | ≡ | 75% | 88% | ≡ |
| **R 0** (%, n) | **88%** | ≡ | - | - | ≡ | 6 | 27 | ≡ |
| **Recurrence** at **R1/Rx** %n | **5%** | 0 | - | - | 0 | 2[1,2] | 1[2] | 0 |
| **HGIEN+Cancer** (n=59) | | - | 8 | 9 | 8 | 1 | 16 | 17 |
| **En bloc** (%, n) | **98%** | - | ≡ | ≡ | ≡ | ≡ | 15 | 16 |
| **R 0** (%) | **93%** | - | 88% | 89% | - | - | 94% | 94% |
| **R curative** (%) | **88%** | - | 75%[3] | 67%[4] | ≡ | ≡ | 94% | 94% |
| **Recurrence** at **$R1_{(h, v)}$** | **3.4%** | - | 1[5] | - | - | - | 1[6] | - |
| **Oncologic Surgery** (%,n) | **6.8%** | - | 1[5] | 2[7] | - | - | 0 | 1[8] |
| **Total** (n=118) **En bloc** | **95%** | | | | | | | |
| **DFS** after ESD/surgery/2nd ESD | **100%** | | ≡ | ≡ | ≡ | ≡ | ≡ | ≡ |

[1]1 adenoma (sm-fibrosis) in the bulbs with h-ESD en bloc in 2 sessions and 1 ESD en-bloc (LGIEN, $R1_h$), [2]HSP +/- APC without recurrence, [3]including one 71 yr-old male with palliatively treated hypopharynx SCC. [4] 3 non-curative ESD. [5]recurrence after diagnostic ESD (R1h) →curative proximal esophagectomy. [6]curative 2nd ESD (G2 pT1b sm1 L0 V0 Pn0; R0). [7]curative distal esophagectomy en bloc for 1 ESD&EID (G2 pT2, R1) and 1 ESD (G2 pT1b-sm2; R0) . [8] diagnostic EID&pmEMR of LST-GN (G2 pT2; R1v) → laparoscopic anterior rectum resection with TME (pTx pN1b[3/12], R0). [5 – 8] see Suppl. Tab. 3.

Abbreviations: **≡ identical**, EID endoscopic intermuscular dissection, HSP hot snare polypectomy, SCC squamous cell carcinoma, SET subepithelial tumor.

## Suppl. Table. S3 Outcome of non-curative ESD-ITT [1]

| Case no.<br>Neoplasm | lesion type 0<br>ESD-ITT[1] | ESD histopathology | R<br>status | management | follow-up (yrs) | | |
|---|---|---|---|---|---|---|---|
| | | | | | DFS | age | status |
| **Esophagus** | | | | | | | |
| # 1<br>**recur. SCC**[2] | IIb<br>ESD | G2 pTis L0 V0 Pn0<br>→ **recur.** after 10 mo | **$R1_h$** | **proximal** (22cm pi)<br>**esophagectomy**<br>pT1a, R0 | 10.7 | 70 | ASA I |
| # 2<br>**SCC** | IIb-**Is**<br>ESD | G2 pT1b **sm2**<br>**L1** V0 Pn0<br>smi **280** µm | R0 | **radiation 45 Gy** | 7.9 | 76 | †<br>prostat.<br>cancer |
| **Barrett esophagus** | | | | | | | |
| # 3<br>**AC** | IIa-**Is**<br>ESD | G2 pT1b **sm2**<br>L0 V0 Pn0<br>smi **729** µm | R0 | only **follow-up,**<br>risk for LNM 5-10% | 9.5 | 82 | †<br>COPD<br>IV |
| # 4<br>**AC** | IIa-**Isp**<br>**ESD** | G2 pT1b **sm2**<br>L0 V0 Pn0<br>smi **856** µm | R0 | **dist. esophagect.**<br>pTx pN0 [0/12], R0 | 12.3 | 66 | ASA I |
| # 5<br>**AC** | IIb+**c** / **Is**<br>ESD & **EID**[3] | G2 **pT2 L1** V0 Pn0 | **$R1_v$** | **dist. esophagect.**<br>pTx pN0 [0/39], R0 | 9.7 | 89 | ASA II |
| **Rectum** | | | | | | | |
| # 6<br>**AC** | LST-GM[4]<br>**ESD** & **EID**[4] &<br>pm-EMR | G2 **pT2 L1** V0 Pn0 | **$R1_v$** | **lap. TME en bloc**<br>pTx pN1b [3/12],<br>R0 | 10.2 | 78 | ASA II |
| **Colon** | | | | | | | |
| # 7<br>**HGIEN** | LST-NGPD<br>1st ESD | **HGIEN**<br>(→ recurr. after 2.3 yr) | **$R1_v$** | **2nd ESD curative**<br>pT1b sm1<br>[150µm]; R0 | 5.6 | 71 | †<br>sepsis,<br>ASA IV |

[1] according to Tab.s 2 in [1,32]. [2] diagnostic ESD in cases #1 - #6.
[2] recurrence after ESD ($R1_h$) of anastomotic recurred SCC (32-34 cm p.i.) after distal esophagectomy→ curative prox. esophagectomy (anastomosis 22 cm p.i.) without recurrence. [3] EID for proper-muscle retracting sign [33]. [4] LST-GM (13x7cm), 3 – 9 cm p.a → ESD&EID(10x6 cm) &pm-EMR → laparoscop. rectum resection with total mesorectal excision (TME) (G2 pN1b [3/12] R0).
Abbreviations: AC adenocarcinoma, DFS disease-free survival, EID endoscopic intermuscular dissection, SCC squamous cell carcinoma, p.i. post incisors. † died from …

## Supplementary Figures

| **Difficulty score for ESD** | | points |
|---|---|---|
| LST-NG or 0-IIc | | 1 |
| Size | 30 – 50 mm | 1 |
| | >50 mm | 2 |
| | ≥ 2/3 circumference | 2 |
| Severe sm fibrosis[1] | | 2 |
| Location | ⬚ | 1 |
| | ▭ | 2 |
| Recurr. after EMR,ESD,OP[2] | | 2 |
| B-II gastric remnant | | 2 |
| **Sum (score)** | | (0-8) |
| **Predicted ESD difficulty:**<br>0 = easy<br>1 = intermediate<br>2-3 = difficult<br>≥4 = very difficult | | |

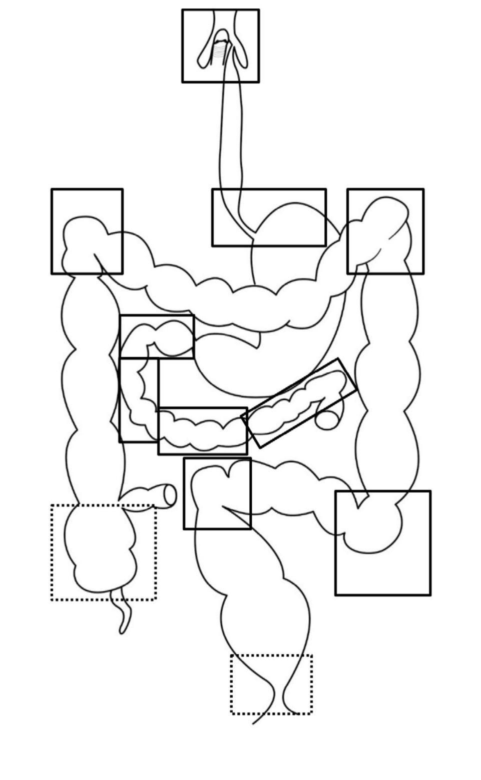

[1] non-lifting or tattoo, [2] at anastomosis

**Suppl Figure. S1. Predictive ESD Difficulty Score for lesion items and anatomical location**, for colorectum acc. to Li B, et al. [30] in exploratory fashion extended to the upper GI tract based on the same scoring items and unfavorable anatomical locations as modified. In colorectum the score predicts difficulty of a planned ESD procedure and correlates best with duration time of ESD.

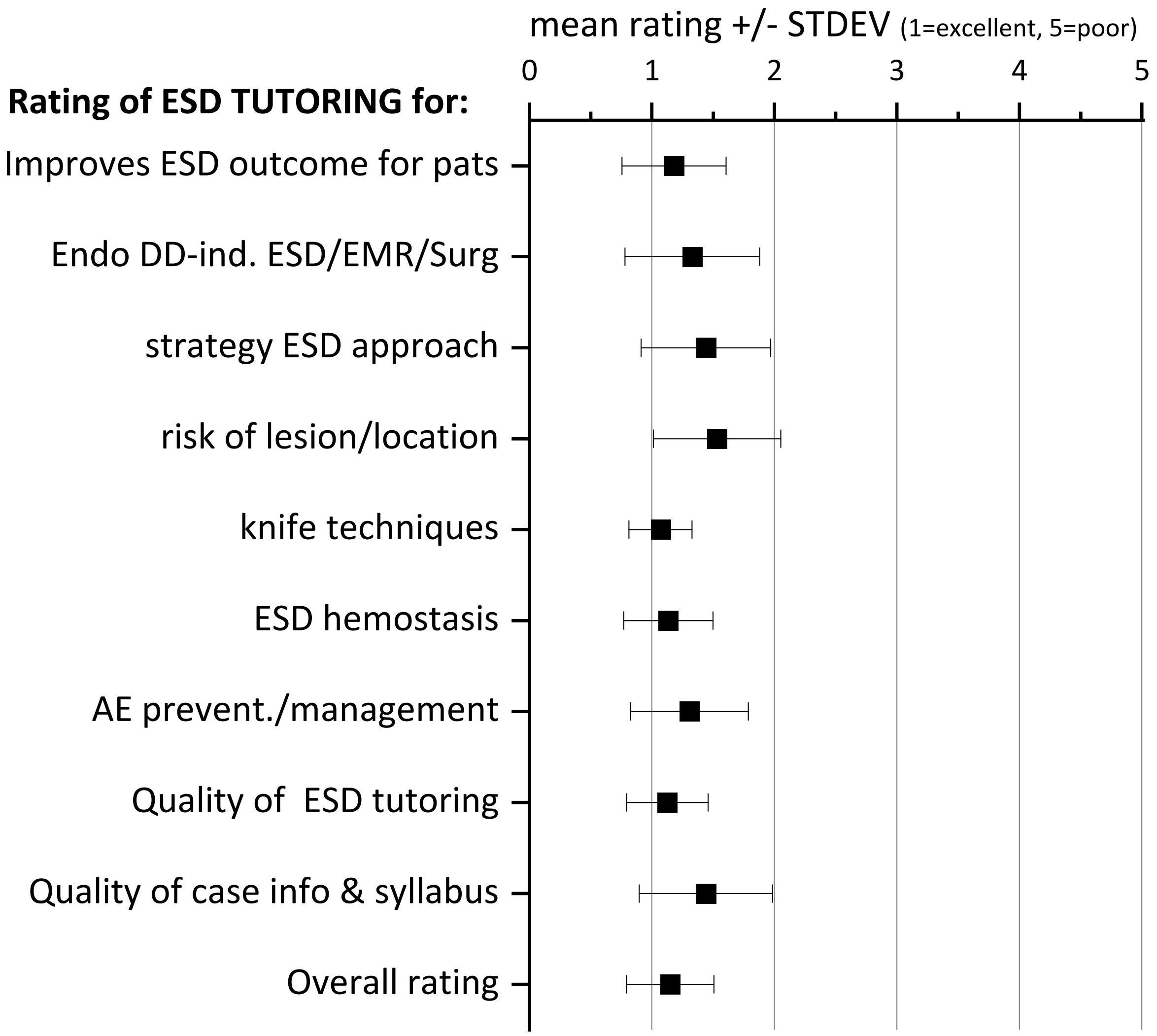


**Suppl Figure. S2: Feedback of ESD implementing endoscopists for the ESD TUTORING program** from 127 feedbacks received (similar for active and passive participants).

## References

1. Oyama, T.; Yahagi, N.; Ponchon, T.; Kiesslich, T.; Berr, F. How to establish endoscopic submucosal dissection in Western countries. *World J Gastroenterol* **2015**, *21*, 11209-11220, doi:10.3748/wjg.v21.i40.11209.
2. Berr, F.; Oyama, T.; Ponchon, T.; Yahagi, N., (Eds.) *Atlas of Early Neoplasias of the Gastrointestinal Tract: Endoscopic Diagnosis and Therapeutic Decisions*. 2 ed.; Springer International Publishing: Cham, SH, 2019.
3. Austrian Medical Practioners Act 1998 (Ärztegesetz 1998), paragraph 36, number 3. Physicians with a foreign place of practice or employment – last update 01.01.2023. Federal Law Gazette (BGBl) I No. 169/1998; European Legislation Identifier (ELI) https://ris.bka.gv.at/eli/bgbl/i/1998/169/P36/NOR40250631.
4. DIRECTIVE 2011/24/EU OF THE EUROPEAN PARLIAMENT AND OF THE COUNCIL of 9 March 2011 on the application of patients' rights in cross-border healthcare. Official Journal of the European Union, L 88/45, Chapter I, Article 1, Chapter III, Articles 7-8. https://eur-lex.europa.eu/LexUriServ/LexUriServ.do?uri=OJ:L:2011:088:0045:0065:en:PDF.
5. The Paris endoscopic classification of superficial neoplastic lesions: esophagus, stomach, and colon: November 30 to December 1, 2002. *Gastrointest Endosc* **2003**, *58*, S3-43, doi:10.1016/s0016-5107(03)02159-x.
6. Ishihara, R.; Inoue, T.; Uedo, N.; Yamamoto, S.; Kawada, N.; Tsujii, Y.; Kanzaki, H.; Hanafusa, M.; Hanaoka, N.; Takeuchi, Y.; et al. Significance of each narrow-band imaging finding in diagnosing squamous mucosal high-grade neoplasia of the esophagus. *J Gastroenterol Hepatol* **2010**, *25*, 1410-1415, doi:10.1111/j.1440-1746.2010.06378.x.
7. Oyama, T.; Inoue, H.; Arima, M.; Momma, K.; Omori, T.; Ishihara, R.; Hirasawa, D.; Takeuchi, M.; Tomori, A.; Goda, K. Prediction of the invasion depth of superficial squamous cell carcinoma based on microvessel morphology: magnifying endoscopic classification of the Japan Esophageal Society. *Esophagus* **2017**, *14*, 105-112, doi:10.1007/s10388-016-0527-7.
8. Muto, M.; Yao, K.; Kaise, M.; Kato, M.; Uedo, N.; Yagi, K.; Tajiri, H. Magnifying endoscopy simple diagnostic algorithm for early gastric cancer (MESDA-G). *Dig Endosc* **2016**, *28*, 379-393, doi:10.1111/den.12638.
9. Oyama, T., (Ed.) *Endoscopic diagnosis of gastric adenocarcinoma for ESD*. Nankodo: Tokyo, 2010.
10. Anagnostopoulos, G.K.; Yao, K.; Kaye, P.; Hawkey, C.J.; Ragunath, K. Novel endoscopic observation in Barrett's oesophagus using high resolution magnification endoscopy and narrow band imaging. *Aliment Pharmacol Ther* **2007**, *26*, 501-507, doi:10.1111/j.1365-2036.2007.03374.x.
11. Oyama, T., (Ed.) *Superficial Barrett´s esophagus carcinoma* Nankodo: Tokyo, 2015.
12. Kudo, S.; Lambert, R.; Allen, J.I.; Fujii, H.; Fujii, T.; Kashida, H.; Matsuda, T.; Mori, M.; Saito, H.; Shimoda, T.; et al. Nonpolypoid neoplastic lesions of the colorectal mucosa. *Gastrointest Endosc* **2008**, *68*, S3-47, doi:10.1016/j.gie.2008.07.052.
13. Sano, Y.; Horimatsu, T.; Fu, K.I.; Katagiri, A.; muto, M.; Ishikawa, H. MAGNIFYING OBSERVATION OF MICROVASCULAR ARCHITECTURE OF COLORECTAL LESIONS USING A NARROW-BAND IMAGING SYSTEM. *Dig Endosc* **2006**, *18*, S44-51.
14. Tanaka, S.; Sano, Y. Aim to unify the narrow band imaging (NBI) magnifying classification for colorectal tumors: current status in Japan from a summary of the consensus symposium in the 79th Annual Meeting of the Japan Gastroenterological Endoscopy Society. *Dig Endosc* **2011**, *23 Suppl 1*, 131-139, doi:10.1111/j.1443-1661.2011.01106.x.
15. Farraye, F.A.; Odze, R.D.; Eaden, J.; Itzkowitz, S.H.; McCabe, R.P.; Dassopoulos, T.; Lewis, J.D.; Ullman, T.A.; James, T., 3rd; McLeod, R.; et al. AGA medical position statement on the diagnosis and

management of colorectal neoplasia in inflammatory bowel disease. *Gastroenterology* **2010**, *138*, 738-745, doi:10.1053/j.gastro.2009.12.037.

16. Inoue, H.; Ikeda, H.; Hosoya, T.; Onimaru, M.; Yoshida, A.; Eleftheriadis, N.; Maselli, R.; Kudo, S. Submucosal endoscopic tumor resection for subepithelial tumors in the esophagus and cardia. *Endoscopy* **2012**, *44*, 225-230, doi:10.1055/s-0031-1291659.
17. Oyama, T.; Tomori, A.; Hotta, K.; Morita, S.; Kominato, K.; Tanaka, M.; Miyata, Y. Endoscopic submucosal dissection of early esophageal cancer. *Clin Gastroenterol Hepatol* **2005**, *3*, S67-70, doi:10.1016/s1542-3565(05)00291-0.
18. Toyonaga, T.; Man, I.M.; Fujita, T.; Nishino, E.; Ono, W.; Morita, Y.; Sanuki, T.; Masuda, A.; Yoshida, M.; Kutsumi, H.; et al. The performance of a novel ball-tipped Flush knife for endoscopic submucosal dissection: a case-control study. *Aliment Pharmacol Ther* **2010**, *32*, 908-915, doi:10.1111/j.1365-2036.2010.04425.x.
19. Yahagi, N.; Fujishiro, M.; Imagawa, A.; Kakushima, N.; Iguchi, M.; Omata, M. Endoscopic Submucosal Dissection for the Reliable en bloc Resection of Colorectal Mucosal Tumors. *Dig Endosc* **2004**, *16*, s89-s92.
20. Oyama, T. Counter traction makes endoscopic submucosal dissection easier. *Clin Endosc* **2012**, *45*, 375-378, doi:10.5946/ce.2012.45.4.375.
21. Oyama, T. Endoscopic Submucosal Dissection for Superficial Esophageal Cancer. In *Endoscopic Submucosal Dissection: Principles and Practice*, Fukami, N., Ed.; Springer New York: New York, NY, 2015; pp. 85-94.
22. Toyonaga, T.; Nishino, E.; Man, I.M.; East, J.E.; Azuma, T. Principles of quality controlled endoscopic submucosal dissection with appropriate dissection level and high quality resected specimen. *Clin Endosc* **2012**, *45*, 362-374, doi:10.5946/ce.2012.45.4.362.
23. Yahagi, N. ESD for Colorectal Lesions. In *Endoscopic Submucosal Dissection: Principles and Practice*, Fukami, N., Ed.; Springer New York: New York, NY, 2015; pp. 103-113.
24. Toyonaga, T.; Ohara, Y.; Baba, S.; Takihara, H.; Nakamoto, M.; Orita, H.; Okuda, J. Peranal endoscopic myectomy (PAEM) for rectal lesions with severe fibrosis and exhibiting the muscle-retracting sign. *Endoscopy* **2018**, *50*, 813-817, doi:10.1055/a-0602-3905.
25. Toyonaga, T.; Man, I.M.; Morita, Y.; Sanuki, T.; Yoshida, M.; Kutsumi, H.; Inokuchi, H.; Azuma, T. The new resources of treatment for early stage colorectal tumors: EMR with small incision and simplified endoscopic submucosal dissection. *Dig Endosc* **2009**, *21 Suppl 1*, S31-37, doi:10.1111/j.1443-1661.2009.00872.x.
26. Berr, F.; Wagner, A.; Kiesslich, T.; Friesenbichler, P.; Neureiter, D. Untutored learning curve to establish endoscopic submucosal dissection on competence level. *Digestion* **2014**, *89*, 184-193, doi:10.1159/000357805.
27. Lauwers, G.Y.; Carneiro, F.; Graham, D.Y.; Curado, M.P.; Franceschi, S.; Montgomery, E.; Tatematsu, M.; Hattori, T. Gastric carcinoma. In *WHO Classification of Tumours of the Digestive System*, Bosman, F.T., Carneiro, F., Hruban, R.H., Theise, N.D., Eds.; WHO Press: Geneva, 2010; pp. 48-58.
28. Schlemper, R.J.; Riddell, R.H.; Kato, Y.; Borchard, F.; Cooper, H.S.; Dawsey, S.M.; Dixon, M.F.; Fenoglio-Preiser, C.M.; Flejou, J.F.; Geboes, K.; et al. The Vienna classification of gastrointestinal epithelial neoplasia. *Gut* **2000**, *47*, 251-255, doi:10.1136/gut.47.2.251.
29. Wagner, A.; Neureiter, D.; Kiesslich, T.; Wolkersdorfer, G.W.; Pleininger, T.; Mayr, C.; Dienhart, C.; Yahagi, N.; Oyama, T.; Berr, F. Single-center implementation of endoscopic submucosal dissection (ESD) in the colorectum: Low recurrence rate after intention-to-treat ESD. *Dig Endosc* **2018**, *30*, 354-363, doi:10.1111/den.12995.
30. Li, B.; Shi, Q.; Xu, E.P.; Yao, L.Q.; Cai, S.L.; Qi, Z.P.; Sun, D.; He, D.L.; Yalikong, A.; Lv, Z.T.; et al. Prediction of technically difficult endoscopic submucosal dissection for large superficial colorectal tumors: a novel clinical score model. *Gastrointest Endosc* **2021**, *94*, 133-144 e133, doi:10.1016/j.gie.2020.11.012.

31. Kantsevoy, S.V.; Wagner, A.; Mitrakov, A.A.; Thuluvath, A.J.; Berr, F. Rectal reconstruction after endoscopic submucosal dissection for removal of a giant rectal lesion. *VideoGIE* **2019**, *4*, 179-181, doi:10.1016/j.vgie.2018.12.001.
32. Pimentel-Nunes, P.; Libanio, D.; Bastiaansen, B.A.J.; Bhandari, P.; Bisschops, R.; Bourke, M.J.; Esposito, G.; Lemmers, A.; Maselli, R.; Messmann, H.; et al. Endoscopic submucosal dissection for superficial gastrointestinal lesions: European Society of Gastrointestinal Endoscopy (ESGE) Guideline - Update 2022. *Endoscopy* **2022**, *54*, 591-622, doi:10.1055/a-1811-7025.
33. Toyonaga, T.; Tanaka, S.; Man, I.M.; East, J.; Ono, W.; Nishino, E.; Ishida, T.; Hoshi, N.; Morita, Y.; Azuma, T. Clinical significance of the muscle-retracting sign during colorectal endoscopic submucosal dissection. *Endosc Int Open* **2015**, *3*, E246-251, doi:10.1055/s-0034-1391665.
